# Reply to Rojas-Ponce, "Operational considerations for implementing culture-free mycobacterial sequencing in routine laboratory settings"

**DOI:** 10.1128/jcm.00564-26

**Published:** 2026-06-12

**Authors:** Kazuki Hashimoto, Kiyoharu Fukushima, Shota Nakamura, Hiroshi Kida

**Affiliations:** 1 Department of Respiratory Medicine and Clinical Immunology, Osaka University Graduate School of Medicine 198269 , Suita, Osaka, Japan; 2 Department of Infection Metagenomics, Research Institute for Microbial Diseases, Osaka University 13013, Suita, Osaka, Japan; 3 Department of Respiratory Medicine, National Hospital Organisation, Osaka Toneyama Medical Center 38519 , Toyonaka, Osaka, Japan; 4 Integrated Frontier Research for Medical Science Division, Institute for Open and Transdisciplinary Research Initiatives, Osaka University 13013, Suita, Japan; 5 Centre for Infectious Disease Education and Research, Osaka University 13013, Suita, Japan; University of Western Australia, Perth, Australia

## REPLY

We thank Dr. Rojas-Ponce for the thoughtful comments on our study comparing culture and culture-free methods for mycobacterial identification (1).

Mucolysis was performed with a semi-alkaline protease at a sputum-to-reagent volume ratio of 1:3, with standardized intermittent mixing on an automated tube mixer (Thermonics THERMO-MIXER TM-137) for 20 min at room temperature—not an operator-dependent manual step. After centrifugation, the pellet was resuspended to a defined final volume of 1 mL in 0.067 mol/L phosphate-buffered saline (Instant Phosphate Buffer 2, PHC Corporation; pH 7.4). The subsequent NALC–NaOH decontamination used the BD MycoPrep kit with a final NaOH concentration of 1.33%, a sediment-to-reagent ratio of 1:2, and an exposure time of 15 min at room temperature. These procedures follow routine clinical-microbiology practice and are implemented at our facility, the National Hospital Organization Toneyama Medical Center. The consistently high accuracy (>90%) in smear-positive specimens supports the reproducibility of this standardized workflow.

We fully agree that pre-analytical factors—pellet recovery, aliquoting, and the physical characteristics of sputum—assume greater importance in low-burden samples, and we explicitly acknowledged in the original report that performance in smear-negative specimens is a principal limitation of the current workflow. Our comparative data reinforce this: acid treatment yielded only 66.7% accuracy versus 90.5% with NALC–NaOH, confirming that preprocessing directly shapes downstream processes. Whether the protease and NALC steps are both strictly necessary or whether the workflow can be streamlined without loss of DNA recovery or decontamination efficiency is an important question that merits prospective evaluation.

An informative analogy comes from bacterial whole-genome sequencing (WGS), which offers strain-level resolution beyond matrix-assisted laser desorption/ionization time-of-flight (MALDI-TOF) mass spectrometry—for example, identifying 89% of anaerobic strains at species level versus 59% by MALDI-TOF and detecting polymicrobial infections missed by mass spectrometry (2)—yet has not supplanted MALDI-TOF in routine clinical microbiology because the principal barriers lie downstream of preprocessing. Beyond pre-analytical optimization, broader implementation experience with pathogen WGS indicates that the dominant barriers to routine adoption lie not in specimen preprocessing—already standardized within clinical microbiology—but in the downstream workflow of library preparation, sequencing, bioinformatics, and clinically actionable reporting (3). We, therefore envisage a core-facility model in which standardized preprocessing is performed at point-of-care hospitals using established protocols, while target-capture sequencing, data analysis, and diagnostic reporting are centralized at regional core facilities. This distributed architecture has already been validated for pathogen WGS, where onsite preprocessing coupled with centralized analytical support outperformed purely centralized laboratory models in both turnaround time and clinical actionability (3), and a nationwide hub-and-spoke precedent exists in the Japanese Initiative on Rare and Undiagnosed Diseases (4). Such a framework directly addresses cost asymmetry, workforce bottlenecks, and turnaround time while preserving reproducibility at the front end.

We endorse Dr. Rojas-Ponce’s call for explicit reporting of processing parameters and validation under routine laboratory conditions, and our group is pursuing multicenter studies, continued preprocessing optimization, and core-facility feasibility evaluations to translate these principles into practice.
